# Correction: Design, synthesis, crystal structure and fungicidal activity of (*E*)-5-(methoxyimino)-3,5-dihydrobenzo[*e*][1,2]oxazepin-4(1*H*)-one analogues

**DOI:** 10.1039/c7md90022h

**Published:** 2017-06-12

**Authors:** Dongyan Yang, Chuan Wan, MengMeng He, Chuanliang Che, Yumei Xiao, Bin Fu, Zhaohai Qin

**Affiliations:** a College of Science , China Agricultural University , Beijing 100193 , China . Email: qinzhaohai@263.net ; Fax: +86 (0)10 62732958 ; Tel: +86 (0)10 62732958

## Abstract

Correction for ‘Design, synthesis, crystal structure and fungicidal activity of (*E*)-5-(methoxyimino)-3,5-dihydrobenzo[*e*][1,2]oxazepin-4(1*H*)-one analogues’ by Dongyan Yang *et al.*, *Med. Chem. Commun.*, 2017, **8**, 1007–1014.



## 

Unfortunately, Scheme 2 showed errors in the structures and substituents. The corrected scheme is shown below:

**Scheme 2:**
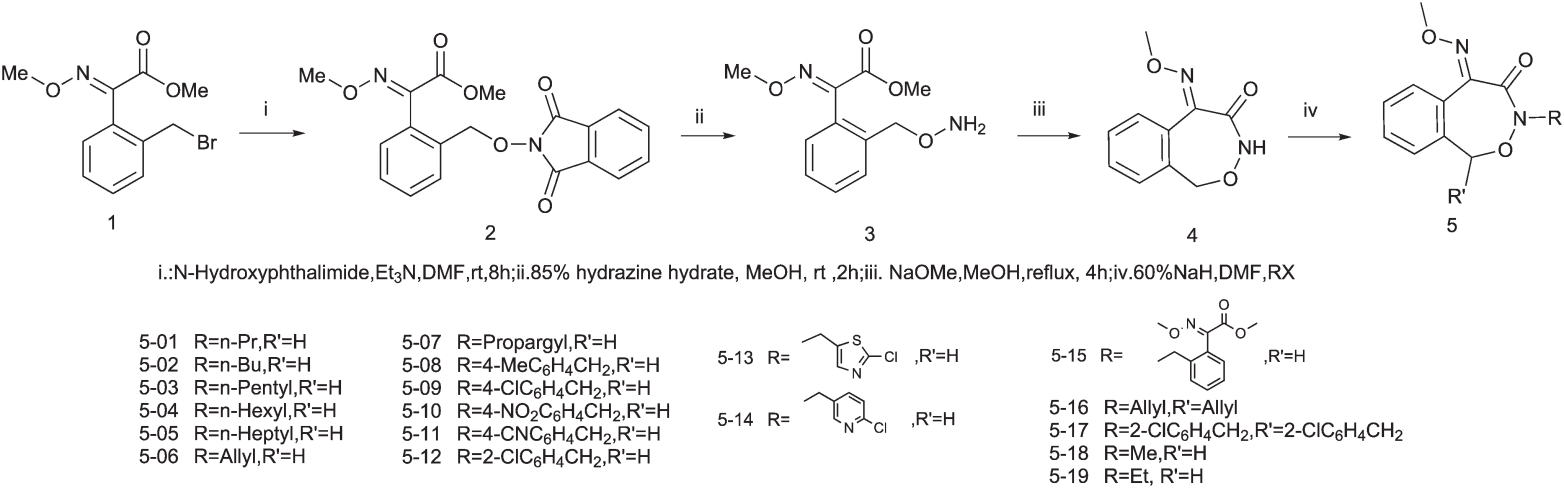


The Royal Society of Chemistry apologises for these errors and any consequent inconvenience to authors and readers.

